# The *Salmonella* Mutagenicity Assay: The Stethoscope of Genetic Toxicology for the 21st Century

**DOI:** 10.1289/ehp.1002336

**Published:** 2010-08-02

**Authors:** Larry D. Claxton, Gisela de A. Umbuzeiro, David M. DeMarini

**Affiliations:** 1 Genetic and Cellular Toxicology Branch, Integrated Systems Toxicology Division, U.S. Environmental Protection Agency, Research Triangle Park, North Carolina, USA; 2 Laboratório de Ecotoxicologia Aquática e Limnologia, Faculdade de Tecnologia, Universidade Estadual de Campinas, Limeira, São Paulo, Brazil

**Keywords:** Ames assay, carcinogenicity, 21st century toxicology, genetic toxicology, high-throughput assays, Salmonella assay, Salmonella mutagenicity assay

## Abstract

**Objectives:**

According to the 2007 National Research Council report *Toxicology for the Twenty-First Century*, modern methods (e.g., “omics,” *in vitro* assays, high-throughput testing, computational methods) will lead to the emergence of a new approach to toxicology. The *Salmonella* mammalian microsome mutagenicity assay has been central to the field of genetic toxicology since the 1970s. Here we document the paradigm shifts engendered by the assay, the validation and applications of the assay, and how the assay is a model for future *in vitro* toxicology assays.

**Data sources:**

We searched PubMed, Scopus, and Web of Knowledge using key words relevant to the *Salmonella* assay and additional genotoxicity assays.

**Data extraction:**

We merged the citations, removing duplicates, and categorized the papers by year and topic.

**Data synthesis:**

The *Salmonella* assay led to two paradigm shifts: that some carcinogens were mutagens and that some environmental samples (e.g., air, water, soil, food, combustion emissions) were mutagenic. Although there are > 10,000 publications on the *Salmonella* assay, covering tens of thousands of agents, data on even more agents probably exist in unpublished form, largely as proprietary studies by industry. The *Salmonella* assay is a model for the development of 21st century *in vitro* toxicology assays in terms of the establishment of standard procedures, ability to test various agents, transferability across laboratories, validation and testing, and structure–activity analysis.

**Conclusions:**

Similar to a stethoscope as a first-line, inexpensive tool in medicine, the *Salmonella* assay can serve a similar, indispensable role in the foreseeable future of 21st century toxicology.

Every day throughout the world, physicians, nurses, and an array of other health professionals use a stethoscope, which was invented by René Laennec in 1816 (Weinberg 1993). It is a relatively simple instrument whose sounds can indicate a myriad of disease states that can then be confirmed by more sophisticated assessments. It is hard to visualize a physician or imagine medicine without the stethoscope. Similarly, the *Salmonella* mutagenicity assay, which was developed initially as a spot test (Ames 1971), then as a plate-incorporation test (Ames et al. 1972) using strains of *Salmonella* bacteria derived from studies by B.N. Ames and P.E. Hartman (Hartman et al. 1986) and rodent liver microsomal activation coupled initially to the assay by H.V. Malling (Malling 1971), is a deceptively simple tool that can be used to detect the mutagenicity of environmental chemicals, environmental mixtures, body fluids, foods, drugs, and physical agents. More complex tests can be applied to confirm and characterize further the mutagenic activity of the agent. Although neither the stethoscope nor the *Salmonella* assay provides a definitive diagnosis/detection of a disease or a mutagen, respectively, both are indispensible first-line tools in their fields.

There is much unrest in the field of toxicology today because of a variety of scientific developments, including advances in genomic science (Parsons et al. 2008; Wood et al. 2007), improved knowledge of the molecular and mechanistic basis for biological responses to toxicant exposure (Guyton et al. 2009), legislation mandating reduced numbers of animals for toxicology testing (Pfuhler et al. 2009), and governmental direction to incorporate all of the above into a new paradigm for toxicology for the 21st century (National Research Council 2007).

A strict parallel cannot be drawn between a targeted testing assay such as the *Salmonella* assay, which is used for hazard identification, and a high-throughput screening (HTS) assay such as either the ToxCast program [U.S. Environmental Protection Agency (EPA)] or the combined U.S. EPA/National Institutes of Health (NIH)/National Institute of Environmental Health Sciences (NIEHS)/National Toxicology Program (NTP) Tox21 program (Kavlock et al. 2009), which can identify specific signaling or biochemical pathways relevant to potential disease development and thus have the possibility of going beyond hazard identification. An assay like the *Salmonella* assay is a stand-alone screen that requires high accuracy and reproducibility and is correlated with health end points, permitting its use for regulatory purposes. In contrast, HTS assays use emerging technologies and target probes, knowledge of biochemical and disease pathways in rodents and humans, genomics, and other technologies to generate a profile or pattern of effects across a range of chemical classes and biological end points that do not depend greatly on any particular chemical or assay result. As with the *Salmonella* assay, HTS assays are viewed as a first-line screening tool, with results of interest being followed up by more extensive confirmatory assays.

In the process of developing and adopting new methods, it is important to build on and learn from past paradigm shifts, several of which occurred in the field of genetic toxicology with the introduction of the *Salmonella* assay. Consequently, the history of the *Salmonella* assay highlights some of the necessary steps and considerations needed for the development of almost any type of toxicology assay, including some aspects of HTS assays. Our purpose with this review is to *a*) describe the paradigm shifts precipitated by the *Salmonella* assay, including the demonstration of a connection between mutagenicity and carcinogenicity and the ubiquitous nature of mutagens in our environment; *b*) document the historic and current applications of the *Salmonella* assay; and *c*) illustrate the lessons learned from the development, validation, testing, assessment, and uses of this *in vitro* assay that may be applicable to the development of *in vitro* toxicology assays for the 21st century.

## Paradigm Shift I: Relating Mutagenic Activity to Carcinogenic Activity

By the middle of the 20th century, there was almost no evidence to support a role for mutation in cancer, and few carcinogens were known to be mutagens (Burdette 1955). However, at this time efforts began to screen carcinogens and other chemicals for mutagenicity *in vitro*, starting with the use of a base-substitution strain of *Escherichia coli* by Demerec et al. (1951) and then by Szybalski (1958), who assessed > 400 compounds using filter-paper disks in a spot test with the same strain. This concept was expanded by the development of a set of tester strains that detected different types of gene mutations in *Salmonella typhimurium* (Whitfield et al. 1966) and in the fungus *Neurospora crassa* (Malling 1966a). Many *in vitro* mutagenicity assays were developed throughout this period, including the *ad-3* forward-mutation assay in *N. crassa* (de Serres and Kølmark 1958); cytogenetic assays (Kihlman 1966); *Hprt* (hypoxanthine-guanine phosphoribosyltransferase) assays in V79 (Chu and Malling 1968) and CHO cells (Hsie et al. 1975); the *Tk^+/−^* (thymidine kinase) assay in mouse lymphoma cells (Clive et al. 1972); and assays in yeast (Zimmermann 1971). The development of these and subsequent assays in mammalian cells and *in vivo* was predicated on the notion that mutagenicity results in these systems would be more relevant to humans than would those from bacteria.

Despite concerted efforts, few mutagens beyond direct-acting alkylating agents were discovered initially with these assays, and known rodent carcinogens other than direct-acting alkylating agents were largely negative in these assays. However, as reviewed by Brusick (1989), a paradigm shift began when Malling (1966b) used a hydroxylating mixture to activate diethyl- and dimethylnitrosamine, which were not mutagenic *in vitro*, to metabolites that were mutagenic in *N. crassa*. Building on this observation, as well as on the work of Miller and Miller (1971) and in consultation with H. Gelboin at NIH/NCI, Malling (1971) then coupled the *Salmonella* mutagenicity assay with *in vitro* metabolic activation composed of the supernatant from mouse liver homogenate centrifuged at 30,000 × *g* (microsomes) plus cofactors. Using this microsomal activation mixture, Malling (1971) showed that dimethylnitrosamine was mutagenic in *Salmonella* in a liquid-suspension assay, resulting in the first version of what would later be called the *Salmonella*/mammalian microsome mutagenicity assay. The host-mediated assay provided additional evidence that carcinogens could be mutagens after mammalian metabolism (Legator and Malling 1971). Ames et al. (1972) then showed that DNA-reactive metabolites of known carcinogens were mutagenic (no metabolic activation was used); in that paper, the authors also introduced the plate-incorporation version of the assay, where the bacteria and chemical were combined in the top agar on the Petri plate.

The connection between mutagenesis and carcinogenesis developed further when Ames et al. (1973a) combined their *Salmonella* tester strains, the test chemical, and the supernatant from a 9,000 × *g* centrifugation of rat liver homogenate (S9 fraction) along with cofactors, as described by Garner et al. (1972), together in the top agar and showed that a variety of heretofore nonmutagenic rodent carcinogens were, in fact, mutagenic after metabolic activation. This plate-incorporation version of the *Salmonella*/mammalian microsome mutagenicity assay became a standard that is still in use today. Various modifications, including reduced nucleotide-excision repair, enhanced cell-wall permeability (Ames et al. 1973b), and enhanced error-prone repair achieved by the introduction of a plasmid [as suggested by MacPhee (1989)], combined to make for a highly sensitive test system (Maron and Ames 1983). Consequently, a new paradigm emerged within just a few years that led to an entirely new approach to carcinogen prediction and testing. This spurred the use of *in vitro* assays for mutagenicity in bacteria and mammalian cells as predictors of potential rodent and human carcinogens (Tennant et al. 1987), culminating in the current genetic toxicity test battery (Eastmond et al. 2009).

Decades of research have shown that mutagenesis is a critical component of carcinogenesis, based on a range of evidence including mutation spectra (Dogliotti et al. 1998; Hainaut and Wiman 2009) and genomic sequencing of tumors (Wood et al. 2007; Parsons et al. 2008). Thus, now it is difficult to recall that once it was somewhat bold to propose that there was any direct connection between the two processes (Knudson 1973; Miller and Miller 1971). Prior to 1972, it was not yet clear that the electrophilicity of some chemical carcinogens had a necessary role in the potential mutagenic activity of such compounds or even that DNA, as opposed to protein, was the ultimate target of carcinogens (Miller 1970).

Although sound theoretical reasons existed for proposing that carcinogens might act through a mutagenic mechanism, a compelling demonstration of this connection did not yet exist (Miller and Miller 1971). In fact, mutagenesis shared the stage with other likely mechanisms, including epigenetic changes (Miller 1970; Miller and Miller 1971), altered expression of an integrated viral genome (Tordaro and Huebner 1972), or alteration of immunological factors by carcinogens, permitting the formation and growth of tumors (Baldwin 1973). Of course, time has shown that all of the above mechanisms are important, especially epigenetic mechanisms (Jones and Baylin 2007), which may be particularly relevant for nonmutagenic carcinogens. Given the much broader range of biology that future assays will detect, new paradigm shifts will emerge in other areas of toxicology from 21st century assays.

## Paradigm Shift II: Recognition of Ubiquitous Mutagenic Activity in the Environment

When Ames (1971) first introduced the assay, he stated “I will be glad to mail the strains to people desiring them and to serve as a clearinghouse for new and improved bacterial tester strains.” Consequently, by the late 1970s, > 2,000 laboratories around the world had requested the *Salmonella* tester strains to initiate studies in environmental mutagenesis (Ames 1979). The fact that neither Ames nor his employer (University of California-Berkeley) patented the strains and that he made them freely available facilitated their use and dissemination throughout academic, industrial, and government laboratories worldwide—promoting the development of many creative uses and modifications of the assay. Creative uses may also emerge from 21st century assays, especially those developed in the public sector, which would have some probability of being disseminated freely.

The initial uses of the *Salmonella* assay led to the startling (at the time) recognition that our environment is replete with mutagens, including fungal toxins, combustion emissions, industrial chemicals, and drugs. The *Salmonella* assay was essential to this effort, providing the means by which researchers discovered for the first time that much of our environment had mutagenic activity, including cigarette smoke (Kier et al. 1974), urban air (Talcott and Wei 1977; Tokiwa et al. 1977), river water (Pelon et al. 1977), drinking water (Loper et al. 1978), food (Sugimura et al. 1977), and soil (Göggelmann and Spitzauer 1983). The assay was used to show that even people could have systemic mutagenic activity detectable in urine after smoking (Yamasaki and Ames 1977) or after eating fried meat (Baker et al. 1982). Decades of studies have shown that nearly all urban air samples tested (Claxton et al. 2004; Claxton and Woodall 2007), drinking water (Richardson et al. 2007), soil (White and Claxton 2004), and house dust (Maertens et al. 2004) are mutagenic. These reviews document that at least 40–50% of the papers published thus far on the genotoxicity of, for example, air, soil, water, and house dust have used the *Salmonella* assay, and they show that the vast majority of contemporary studies rely almost exclusively on the *Salmonella* assay for mutagenicity assessments of environmental media.

The realization that much of the environment had mutagenic activity was unanticipated by most researchers and posed a challenge to environmental scientists, public health authorities, and regulators. As 21st century toxicology proceeds, previously unrecognized, ubiquitous toxicities in our environment may be discovered—beyond findings of mutagenicity and potential carcinogenicity—and a new paradigm of toxicity effects may emerge (Boekelheide and Campion 2010). Regulators and public health authorities may have to expand or reconsider their approaches based on the results from such assays.

## How the *Salmonella* Assay Has Been Used

### Published data

We searched three publication databases [PubMed (http://www.ncbi.nlm.nih.gov/pubmed/), Scopus (http://www.scopus.com/home.url), and Web of Knowledge (http://apps.isiknowledge.com/)], and we found 10,169 unique publications dealing with the *Salmonella* assay. This was accomplished by searching each database for “Ames test OR *Salmonella* mutagen.” This gave 11,064 responses in PubMed, 13,694 in Scopus, and 3,453 in the Web of Knowledge. Although it is likely that not all references were found in this search, the number of references retrieved should give a good sampling of trends. We merged the citations into an EndNote (Thomson Reuters, New York, NY) database, and we deleted duplicates based on the same first author name, journal name, journal year, volume, and page number. We examined the remaining information individually to eliminate additional duplicates, non-*Salmonella* mutagenicity papers, abstracts, and papers that seemed to refer to the assay only tangentially. Then we categorized papers by key words/phrases that reflected how the assay was used or discussed within the context of the paper. The final database had 10,169 publications sorted into 7 major categories and 20 subcategories. A publication was often included in more than one category/subcategory based on the nature of that publication. The reference database is available in Supplemental Material (doi:10.1289/ehp.1002336).

**Figure 1:**
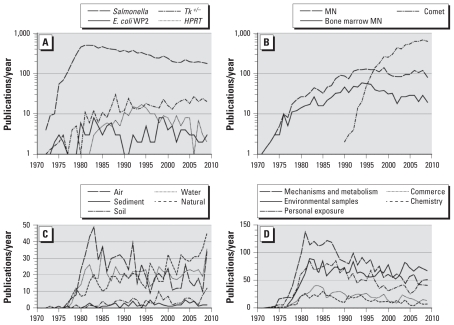
Peer-reviewed journal articles published per year for genetic toxicology bioassays. (*A*) Publications for the classical gene mutation assays [*Salmonella* assay, *E. coli* mutagenicity assays (*E. coli* WP2), *Hprt* assays in V79 and CHO cells or *HPRT* in TK6 cells (*HPRT*), and the mouse lymphoma *Tk^+/−^* assay. (*B* ) Publications for all micronucleus (MN) assays, bone-marrow MN, and comet assays. (*C*) Publications for the *Salmonella* assay involving environmental substances (air, water, natural products, soil, and sediments). (*D*) Publications for the *Salmonella* assay involving various categories of studies (mechanisms and metabolism, environmental samples, personal exposure studies, commerce, and chemistry). The reference database is available in Supplemental Material (doi:10.1289/ehp.1002336).

Figure 1A shows the numbers of publications per year that have used the *Salmonella* assay as well as the other gene-mutation assays developed near the same time, including those in mammalian cells. The number of publications using the *Salmonella* assay rose dramatically, peaking at approximately 500 papers/year in the early 1980s, but has declined gradually to a rather constant level of approximately 200 papers/year during the past decade. Other assays rose to approximately 10–20 papers/year, with the mouse lymphoma *Tk^+/−^* assay remaining at that level today.

Subsequently, newer genotoxicity assays became popular, and the number of publications for these are shown in Figure 1B. By far, the comet assay has the highest surge in usage and is just now starting to plateau. The micronucleus assays also are prominent, with approximately 100 papers/year being published consistently for the past 20 years. The publication frequency for papers using micronucleus assays has surpassed those using *in vitro* chromosome aberration assays, which peaked in the mid-1980s (data not shown).

With regard to the *Salmonella* assay, the papers documenting the testing of agents associated with environmental samples (Figure 1C), as well as commerce, metabolism, or personal exposure (Figure 1D), peaked in the 1980s but still continue at a steady rate. A closer look at the number of papers published on various types of environmental samples (Figure 1C) shows that *a*) relatively few publications have been associated with soil and sediment samples; *b*) papers looking at air samples follow the overall declining trend seen since 1983; and *c*) publications dealing with water reached a plateau starting in 1980 and have remained stable. However, reports dealing with natural substances have increased since the mid-1990s. This increase is due largely to a search for and analysis of antimutagens, mainly from plant extracts. Figure 1D shows a decline in the number of publications on mechanism and metabolism; although there was a rise in the personal-exposures subcategories until the late 1980s (Figure 1D), the number has since declined.

### Unpublished data

For a variety of reasons, little toxicological data have either been generated or are available publically for a large proportion of compounds in commercial use. For example, toxicological data are available for only 7% of high-production-volume chemicals (> 1 million pounds/year) (Guth et al. 2007) and for only a fraction of regulated industrial chemicals (Schwarzman and Wilson 2009; Wilson and Schwarzman 2009). The few publications dealing with commercial substances (Figure 1D) likely reflect the fact that such data are proprietary. In the U.S. EPA New Chemicals Program, approximately 50,000 premanufacturing notice (PMN) cases have been received since 1979 when the program began; however, only 10% (4,997) have mutagenicity data, with 87% of these (4,351) having *Salmonella* assay data (Cimino MC, personal communication). Thus, only 8.7% of the 50,000 PMNs submitted during the past 30 years have *Salmonella* mutagenicity data, almost none of which are available publically, and approximately 2% of pre-1979 PMNs have been reviewed for the need for toxicological data (Guth et al. 2007).

To estimate the percentage of commercial chemicals that are mutagens, Zeiger and Margolin (2000) assembled randomly 100 chemicals in commercial use, which included 46 organics in highest production in the United States (inorganic and elemental compounds were not included among the 100 chemicals), and evaluated them for mutagenicity in the *Salmonella* assay. They found that 22% of the total 100 compounds were mutagenic, and 20% of the subset of 46 high-production compounds were mutagenic. In the absence of required testing and reporting (Guth et al. 2007; Schwarzman and Wilson 2009), these data are the best estimates available regarding the proportions of mutagens among organic compounds in current commercial use. Improved estimates may emerge after potential changes to the Toxic Substances Control Act (TSCA) (Birnbaum 2010; U.S. EPA 2010b; Wilson and Schwarzman 2009).

The U.S. Food and Drug Administration (FDA) Center for Drug Evaluation and Research (CDER) program does not keep cumulative data for each assay submitted, largely because each submission is usually considered solely on the basis of the information within it (Benz RD, personal communication). It must be assumed, however, that the pharmaceutical industry also has tested thousands of substances in the *Salmonella* assay. In an analysis using the *Physicians’ Desk Reference* from 1999 through 2008, Snyder (2009) compiled the mutagenicity of > 500 marketed drugs, excluding the cytotoxic anti-cancer and antivirals, nucleosides, steroids, and biologicals. He found that approximately 7% (38/525) of these drugs were mutagenic in bacterial assays (data from either *E. coli* or *Salmonella* assays); this small percentage is likely due to the extensive early screening in the *Salmonella* assay to eliminate mutagenic molecules from further development.

There are a few reports of environmental monitoring programs using the *Salmonella* assay, such as the 20-year program on surface-water mutagenicity in Brazil (Umbuzeiro et al. 2001). However, such monitoring is rarely done and almost never reported in the peer-reviewed literature, although the Brazilian data are available online from the Companhia Ambiental do Estado de São Paulo (CETESB) (2010). Therefore, the large number of agents whose test results in the *Salmonella* assay have been published may not reflect either the equally large—or larger—number of proprietary chemicals tested by the pharmaceutical and chemical industries or environmental monitoring data, which are not published.

## The *Salmonella* Assay as a Model for 21st Century Toxicology Assays

Because of its simplicity, cost effectiveness, flexibility, and large validated database, the *Salmonella* assay is an ideal model to consider in the development of equally reliable *in vitro* toxicology assays for the 21st century. The predictivity, specificity, and sensitivity of the *Salmonella* assay have been validated against selected other mutagenicity assays and rodent carcinogenicity data (Tennant et al. 1987). Likewise, new HTS assays will need to be validated against something (Hartung 2009a), and one possibility is to measure some end points against the *Salmonella* assay (Schoonen et al. 2009). As outlined by Zeiger (2003), there are fundamental procedures to consider when developing, validating, and ultimately accepting new assays, and below we highlight some ways in which the *Salmonella* assay serves as a model for this process.

### Standard procedures, quality assurance, and statistical assessment

Soon after the introduction and widespread use of the *Salmonella* assay, researchers recognized the need for standardized procedures. Consequently, Ames published methods papers (Ames et al. 1975; Maron and Ames 1983), and the procedures were quickly adopted by the mutagenesis community. Procedures included the use of positive and negative controls, standard procedures for performing the assay, preparation of S9 mix, checking the tester strains for genetic and physiological stability, and evaluating the results statistically (Bernstein et al. 1982; Claxton et al. 1984, 1987; Kim and Margolin 1999; Margolin et al. 1981; McCann et al. 1984; Stead et al. 1981). Although positive controls and metabolic activation were generally missing from some first-generation HTS assays, these and other issues are being considered and corrected in current and future iterations of the ToxCast and Tox21 assays (Hartung 2009a, 2009b; Huang et al. 2009; Kavlock et al. 2008; Westerink et al. 2010), as well as for toxicogenomic assays (Ellinger-Ziegelbauer et al. 2009). As noted above, even the early versions of the *Salmonella* assay did not incorporate metabolic activation (because it had not yet been developed). Despite these limitations, initial analyses of data from ToxCast Phase 1 have identified those chemicals able to induce oxidative stress as evidenced by Nrf2 activity (Martin et al. 2010).

### Structure–activity analysis (SAR)

Data from the *Salmonella* assay were used by Ashby (1985) to identify structural alerts for potential carcinogenicity, providing critical data for the development of computerized structure–activity methods for carcinogenicity prediction (Richard 1998). These methods are still used widely within the chemical, pharmaceutical, and regulatory communities (Benfenati et al. 2009). Claxton et al. (1988) examined *Salmonella* assay data in the peer-reviewed literature for individual chemicals, classified the chemicals by an International Union of Pure and Applied Chemistry chemical class scheme, and found that mutagenicity in the *Salmonella* assay was highly predictive of rodent carcinogenicity for some chemical classes, such as aromatic amines, polycyclic aromatic hydrocarbons, and nitroarenes, but was less predictive for others, such as chlorinated organics. Ashby and Tennant (1988) noted that for 222 chemicals evaluated by the NTP, data from the *Salmonella* assay, combined with structural alerts and a more limited protocol for the rodent cancer bioassay, permitted the detection of trans-species/multiple-site rodent carcinogens, which are likely human carcinogens (Ashby and Paton 1993; Tennant 1993).

Building on this past success, current efforts still rely on *Salmonella* assay data and are extending the analyses using newly developed computational methods and structural features. For example, Hansen et al. (2009) assembled a benchmark database containing 6,500 chemicals with *Salmonella* assay data along with structural information [Simplified Molecular Input Line Entry Specifications (SMILES)] to develop a prediction model that outperforms a variety of commercial predictive tools. Yang et al. (2008) compiled a group of 2,428 compounds, each of which has structural information and data for six mutagenicity tests, and showed that the percentage of industrial chemicals that were mutagenic was greater than that of chemicals used as drugs or food ingredients. The incorporation of chemical structure into the DSSTox EPA ToxCast continues to grow (Houck et al. 2008), and this structural and toxicology database will enable data from HTS assays to be used for SAR as *Salmonella* assay data have been used for decades.

### Reproducibility and transferability of the assay across laboratories

High reproducibility of an assay allows results to be compared not only within the same laboratory over time but also among laboratories. To address this issue, a set of international, collaborative testing programs was established to evaluate the *Salmonella* assay as well as several other mutagenicity assays using coded chemicals from the same lot (Ashby et al. 1985, 1988; de Serres et al. 1981) and standard protocols (Dunkel et al. 1984, 1985; Margolin et al. 1984; Piegorsch and Zeiger 1991). These comparative studies paved the way for the establishment of standard methods and procedures for selected mutagenicity assays that are still largely in place. A similar international effort was established for the evaluation of standards of complex mixtures in the *Salmonella* assay (Claxton et al. 1992; Lewtas et al. 1992).

Concurrently, the establishment of the U.S. EPA GENE-TOX program (Ray et al. 1987; Waters and Auletta 1981) provided, to our knowledge, the first self-assessment of the literature in any field of toxicology—in this case, genetic toxicology. This enormous effort (Waters 1994) involved 196 scientists who critically read all of the papers published on each of 23 assays, resulting in 41 comprehensive, published reviews. The consequence of this effort was that out of nearly 200 assays, the mutagenesis community agreed on the general use of a subset for routine use, including, for example, the protocols, publication requirements, and use of positive and negative controls, much of which is reflected in the current genotoxicity test battery (Eastmond et al. 2009).

As a plethora of new assays emerge over the coming years, a similar self-assessment being organized by the Transatlantic Think Tank of Toxicology (Hartung 2009a) will be invaluable. Just as with the self-assessment by the GENE-TOX program, it will likely result in the acceptance of just a few assays, as well as the establishment of the standards, protocols, interpretation, and publication requirements for those assays, which will provide a test battery that will serve the regulatory community well in the coming years.

### Testing

As reviewed by Zeiger (2004), many factors led to the initial effort of the U.S. government, in particular, M. Legator at the FDA, to sponsor mutagenicity testing in 1971, followed by numerous contracts in the ensuing years. Ames himself published an extensive testing and validation study early on in which he used his assay to assess the mutagenicity of 300 compounds (McCann et al. 1975; McCann and Ames 1976). This effort was followed soon by other screening studies involving the *Salmonella* and other assays (Bruce and Heddle 1979; Ishidate and Odashima 1977; Nagao et al. 1978; Purchase et al. 1978; Rinkus and Legator 1979). The NIEHS/NTP mounted the most comprehensive effort in testing, involving the comparison of four mutagenicity assays along with rodent carcinogenicity data (Tennant et al. 1987). This effort and subsequent analyses (Kirkland et al. 2005; Zeiger 1998) have shown that the *Salmonella* assay alone, in the absence of a test battery, is reasonably predictive of rodent carcinogenicity. Among a group of chemicals of mixed chemical class, a greater percentage of the compounds that are mutagenic in the *Salmonella* assay are likely to be rodent carcinogens compared with the percentage of nonmutagens likely to be noncarcinogens (Kirkland et al. 2005; Zeiger 1998). At present, there are no reliable methods to assess *Salmonella*-negative compounds for potential carcinogenicity. This conclusion has prompted discussion, pro and con, regarding the option of eliminating the mammalian cell assays from the genotoxicity test battery or the inclusion of other assays (Elespuru et al. 2009; Kirkland et al. 2007).

This development is ironic, as efforts proceed swiftly to develop high-throughput assays in mammalian cells (Kavlock et al. 2008; Westerink et al. 2010). Despite the theoretical and scientific relevance of mammalian cell assays, their prognostic value may, in fact, be limited. For example, the *Salmonella* assay is less susceptible than mammalian cell assays to artifacts resulting from high toxicity, pH shifts, and osmotic effects (Kirkland et al. 2007). Nonetheless, Zhu et al. (2008) showed that using HTS cell viability data for 1,408 compounds greatly improved quantitative structure–activity relationship (QSAR) predictions for rodent carcinogenicity. They suggest that an approach using improved models, coupled with HTS assay data and structural features of the compounds, might partially replace *in vivo* toxicity testing. Even some *in vivo* assays may be of little or no added value, as indicated by the inability of the mouse bone-marrow micronucleus assay to improve carcinogen prediction beyond that of the *Salmonella* assay alone (Zeiger 1998).

The history of genetic toxicology demonstrates that only assays that can be adopted by many laboratories and validated through extensive testing are of value for regulatory purposes. Consequently, based on the testing efforts described above, testing schemes were put into law for testing new chemicals (U.S. EPA 2010b), pesticides (U.S. EPA 2010a), and new pharmaceuticals (FDA 2010). Recent discussions have explored how new types of assay data might have an impact on the regulation of genotoxic compounds (Elespuru et al. 2009; Ge et al. 2007; Guyton et al. 2009; Hartung 2009a, 2009b; Hartung and Daston 2009; Hartung and Rovida 2009; Hoppin and Clapp 2005; Krewski et al. 2009; Meek and Doull 2009; National Research Council 2007; Service 2009). Many such issues will need to be settled before legislation of the type above could ever be instituted for 21st century assays.

### Assay flexibility

The flexibility of the *Salmonella* assay has allowed the assay to be used in a variety of protocols with a variety of agents, including complex mixtures, gases, and radiation. Current HTS assays use nonvolatile, single agents that are soluble in dimethyl sulfoxide, but agents with other characteristics (e.g., water-soluble compounds, gases) will need to be tested (Kavlock et al. 2008; Tice RR, personal communication). Over the years, this recognition for the *Salmonella* assay resulted in a plethora of modifications that have enabled the assay to be used in an almost infinite variety of ways. These include modifications permitting *a*) the use of small amounts of sample (Diehl et al. 2000; Flamand et al. 2001; Green et al. 1977; Houk et al. 1989; Kado et al. 1983) in semi–high-throughput modes involving colorimetric analysis (Kamber et al. 2009; Umbuzeiro et al. 2010) and fluorescent assays (Aubrecht et al. 2007; Cariello et al. 1998); *b*) the testing of volatiles and gases (Baden et al. 1976; Hughes et al. 1987); *c*) the testing of body fluids, including urine (Cerná and Pastorková 2002), feces (de Kok and van Maanen 2000), breast milk (Phillips et al. 2002; Thompson et al. 2002), breast nipple aspirates (Klein et al. 2001), and cervical mucus (Holly et al. 1993); *d*) the testing of all types of complex mixtures, including air, soil, water, house dust, and combustion emissions (see “Paradigm Shift II” above), and fried meat (Knize and Felton 2005); *e*) molecular (DeMarini 2000; Koch et al. 1994) and genomic analyses (Porwollik et al. 2001; Ward et al. 2010); and *f*) the evaluation of mutagenicity inside the International Space Station (Rabbow et al. 2003). This flexibility has permitted the *Salmonella* assay to be used for almost every conceivable type of environmental and molecular epidemiology study.

In addition, numerous modifications of the tester strains or testing conditions have permitted researchers to explore the role of metabolism and to detect the mutagenicity of specific chemical classes of substances (Claxton and Barnes 1981; Gee et al. 1994; Hagiwara et al. 1993; Hayashida et al. 1976; Houk and Claxton 1986; Houk et al. 1989; Josephy 2002; Prival and Mitchell 1982; Reid et al. 1984; Rosenkranz and Mermelstein 1983; Watanabe et al. 1990). Whether it has been in the development of commercial products (Zeiger and Margolin 2000), the evaluation of industrial products and wastes (Aguayo et al. 2004; Bessi et al. 1992; Brooks et al. 1998; Claxton et al. 1998; Ohe et al. 2004), or substances known to contaminate the environment (Chen and White 2004; Claxton et al. 2004; Claxton and Woodall 2007; White and Claxton 2004), the *Salmonella* assay has been the screening test of choice in genetic toxicology for nearly four decades. Perhaps a new assay will emerge in the coming years that can assess a comprehensive set of predictive biological changes and also have the range of flexibility exhibited by the *Salmonella* assay.

### Standardization of sample preparation

The flexibility of the *Salmonella* assay prompted the development of methods to prepare environmental samples for the assay (Hewitt and Marvin 2005; Marvin and Hewitt 2007). This included solvents and materials for the delivery of substances to the assay, preparation of environmental and epidemiological samples, and methods for the concentration and determination of doses for testing gases. The coupling of chemical methods with the *Salmonella* assay enabled extensive use of the assay for bioassay-directed chemical fractionation to identify chemical classes of mutagens or individual mutagens (Austin et al. 1985; Brooks et al. 1998; Lewtas 1993; Lewtas et al. 1990; Oliveira et al. 2006), permitting the discovery of many environmental mutagens, such as PBTA (2-phenylbenzotriazole) in surface waters (Nukaya et al. 1997), MX (3-chloro-4-(dichloromethyl)-5-hydroxy-2(5*H*)-furanone) in drinking water (Hemming et al. 1986), and PhIP (2-amino-1-methyl-6-phenylimidazo[4,5-*b*]pyridine) in fried meat (Felton et al. 1986). The Tox21 program is already testing herbal agents and has plans to test complex mixtures and environmental samples (Tice RR, personal communication). Coupled with bioassay-directed fractionation, this effort could provide new opportunities for identifying environmental hazards and characterizing health effects from environmental pollution.

## Conclusions

If the *Salmonella* assay can be likened to the stethoscope, then ample studies have confirmed repeatedly the invaluable role that the *Salmonella* assay alone plays in identifying rodent (Kirkland et al. 2005; Yang et al. 2008; Zeiger 1998) and human (Morita et al. 1997) carcinogens. A physician may not make a final diagnosis based solely on the sounds heard through the stethoscope, but in many cases, such sounds prove to be invaluable in formulating the confirmatory procedures. Perhaps some of the emerging HTS (Kavlock et al. 2008), toxicogenomic (Ellinger-Ziegelbauer et al. 2009), and short-term rodent assays (Jacobson-Kram 2010) can be likened to the cardiology methods that would be used to follow up anomalies detected by the stethoscope of genetic toxicology, i.e., the *Salmonella* assay.

Because of its historical database, intrinsic value, flexibility, and low cost, the *Salmonella* assay will not soon be replaced for the hazard identification of new chemicals or environmental samples. Indeed, chemicals whose annual production exceeds 1 ton/year (~ 30,000 compounds) are scheduled to be tested in the *Salmonella* assay under the European Union’s Registration, Evaluation, Authorization, and Restriction of Chemicals (REACH) legislation (Poth and Jaeger 2007). Experience with the *Salmonella* assay should serve as a model for the development and deployment of new approaches to predict and understand the toxicology of substances. The use of the *Salmonella* assay may not be as lasting as that of the stethoscope, but the *Salmonella* assay has made a significant mark on the history of toxicology and has an indispensable role to play in the foreseeable future of 21st century toxicology.
